# Relationship Between Left Atrial Strain and Atrial Fibrillation: The Role of Stress Echocardiography

**DOI:** 10.3390/diagnostics15010007

**Published:** 2024-12-24

**Authors:** Alfredo Mauriello, Adriana Correra, Antonia Ascrizzi, Gerardo Elia Del Vecchio, Giovanni Benfari, Federica Ilardi, Matteo Lisi, Alessandro Malagoli, Giulia Elena Mandoli, Maria Concetta Pastore, Simona Sperlongano, Matteo Cameli, Vincenzo Russo, Antonello D’Andrea

**Affiliations:** 1 Cardiology Unit, Department of Medical and Translational Sciences, University of Campania “Luigi Vanvitelli”, “V. Monaldi” Hospital, 80131 Naples, Italy; alfredo.mauriello93@libero.it (A.M.); antonia.ascrizzi@gmail.com (A.A.); delvecchiogerardo@gmail.com (G.E.D.V.); simona.sperlongano@unicampania.it (S.S.); vincenzo.russo@unicampania.it (V.R.); 2 Cardiology and Intensive Care Unit, Department of Cardiology, “Umberto I” Hospital, 84014 Nocera Inferiore, Italy; 3 Intensive Cardiac Care Unit, “San Giuseppe Moscati” Hospital, ASL Caserta, 81031 Aversa, Italy; adrianacorrera@gmail.com; 4 Section of Cardiology, Department of Medicine, University of Verona, 37100 Verona, Italy; giovanni.benfari@univr.it; 5 Department of Advanced Biomedical Sciences, Division of Cardiology, Federico II University Hospital, 80131 Naples, Italy; federica.ilardi@unina.it; 6 Department of Cardiovascular Disease—AUSL Romagna, Division of Cardiology, Ospedale “S. Maria delle Croci”, 48121 Ravenna, Italy; matteo.lisi@hotmail.it; 7 Division of Cardiology, Nephro-Cardiovascular Department, “Baggiovara” Hospital, 41100 Modena, Italy; ale.malagoli@gmail.com; 8 Department of Medical Biotechnologies, Division of Cardiology, University of Siena, 53100 Siena, Italy; giulia_elena@hotmail.it (G.E.M.); pastore2411@gmail.com (M.C.P.); matteo.cameli@unisi.it (M.C.)

**Keywords:** speckle tracking, strain, atrial fibrillation, echocardiography, stress echocardiography

## Abstract

Interest in advanced echocardiographic imaging methods is growing. Left atrial strain (LAS) is among recently developed echocardiographic parameters. LAS represents an index of tissue deformation of the left atrium (LA). This parameter is an expression of LA function. Several arrhythmias are caused by impaired LA function. LAS can be assessed with a resting echocardiogram. The evaluation of LAS during stress echocardiography represents another model for assessing LA function. The development of altered LAS during physical or pharmacological stress is a predictor of early LA disease. Our review aims to evaluate the relationship between alterations in LAS and the development of atrial fibrillation (AF), and the diagnostic and prognostic roles of the stress echocardiogram in clinical practice.

## 1. Introduction

Atrial fibrillation (AF) is the most common sustained cardiac arrhythmia, with significant implications for morbidity, mortality, and healthcare costs. AF is a supraventricular arrhythmia characterized by uncoordinated electrical activity in the atria, leading to a loss of effective atrial contraction. The surface electrocardiogram (ECG) shows the absence of regular and discernible P waves and irregular ventricular activation [1]. Clinical presentation may vary from completely asymptomatic patients to severe acute heart failure (HF), and could include several complications, particularly cerebral and systemic embolic events. Thus, the early detection and management of AF, as well as associated structural and functional changes in the atria, are critical to improving clinical outcomes [2].

Multimodality imaging techniques should be used individually for each patient presenting with AF [3]. Echocardiography has a central role in AF management and has a class I recommendation for guiding treatment decisions, as it is an invaluable tool in all the four domains of the AF-CARE approach suggested by the latest European guidelines: Comorbidity and risk factor management; Avoiding stroke and thromboembolism; Reducing symptoms through rate and rhythm control; and Evaluation and dynamic reassessment [1]. Traditional echocardiographic techniques, although valuable, may be limited in their ability to assess subtle atrial dysfunction, particularly in the context of AF. In this regard, echocardiographic atrial strain imaging has emerged as a promising tool for evaluating atrial function with greater sensitivity, allowing for early detection of individuals with high risk of AF incidence or recurrence and identification of those exposed to increased risk of stroke and systemic thromboembolism, even when in sinus rhythm [4,5,6].

Atrial strain, derived from speckle tracking echocardiography (STE), provides a quantitative assessment of atrial deformation and reservoir function, reflecting atrial compliance, contractility, and overall performance [7,8]. Hence, this technique is crucial for evaluating diastolic function and the characterization of so-called “atrial myopathy” [9,10]. This advanced imaging modality has shown potential not only in resting conditions but also under stress, where the hemodynamic demands on the heart are altered [11,12]. Exercise and pharmacological stress echocardiography (SE) have a pivotal role in detecting diastolic dysfunction and in the diagnostic algorithm of HF with preserved ejection fraction (HFpEF), conditions that are strictly linked to AF [13,14,15]. Therefore, assessing left atrial strain (LAS) during SE might add important information, since it can unmask latent atrial dysfunction that may not be evident at rest, offering further insights into the burden of AF and the risk of its recurrence.

This review explores the evolving role of atrial strain imaging, particularly in the context of exercise and pharmacological SE, and its clinical relevance in assessing and managing patients with AF. The integration of atrial strain into SE protocols holds promise for improving the detection of atrial dysfunction, refining risk stratification, and guiding therapeutic strategies in this challenging population.

## 2. Speckle Tracking and Left Atrial Strain

**Figure 1:**
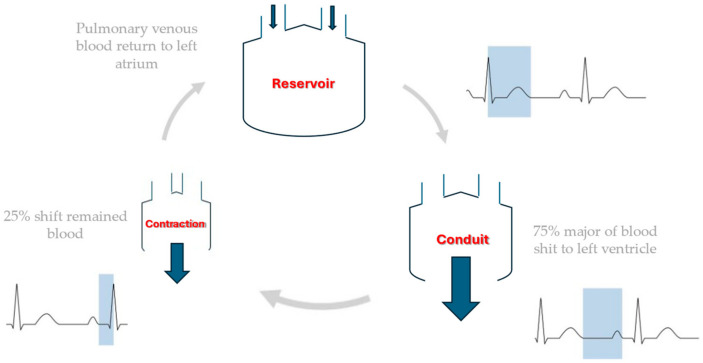
Different phases of the LA during the cardiac cycle.

**Figure 2:**
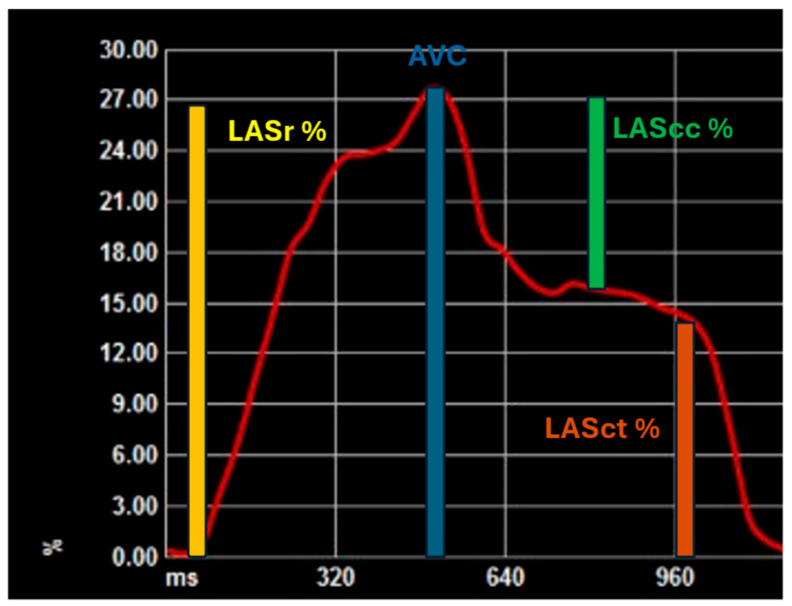
Different phases of LAS during the cardiac cycle. AVC—aortic valve closure; LAScc—left atrial strain conduction; LASct—left atrial strain contraction; LASr—left atrial strain reservoir.

STE represents a significant advancement in cardiac imaging, offering a non-invasive and highly accurate method for assessing left atrial (LA) mechanical function. This technique provides a comprehensive evaluation of LA function, encompassing the three primary phases of atrial activity: reservoir, conduit, and contractile [16,17,18]. During the reservoir phase, reservoir strain reflects the LA’s capacity to store blood during ventricular systole, as the atrium fills with blood returning from the pulmonary veins. During the reservoir phase, left ventricular function should also be assessed. In fact, the reservoir phase is also influenced by left ventricular contractility, left atrial compliance and the degree of mitral regurgitation. The increase in left atrial strain reservoir (LASr) during exercise is due to an increase in left ventricular contractility and the increase in left atrial stiffness is thought to result in a reduction in the normal value. Therefore, if the contractile reserve of the left ventricle is reduced, the increase in LASr will be reduced regardless of the properties of the left atrium. Therefore, in order to assess LASr during exercise, it is necessary to assess the contractile function of the left ventricle at the same time [19]. The conduit phase, assessed by conduit strain, occurs in early diastole. During this phase, the atrium acts as a passive conduit, allowing blood to flow from the pulmonary veins into the left ventricle in preparation for the next contraction [19]. Lastly, the booster pump phase occurs during late diastole, when the atrium contracts to provide additional blood flow into the left ventricle. The booster strain phase is of particular importance in ensuring that the left ventricle has an adequate preload, particularly during periods of increased physiological demand [19]. Figure 1 and Figure 2 represent different phases of LA function and LAS during the cardiac cycle.

**Figure 3:**
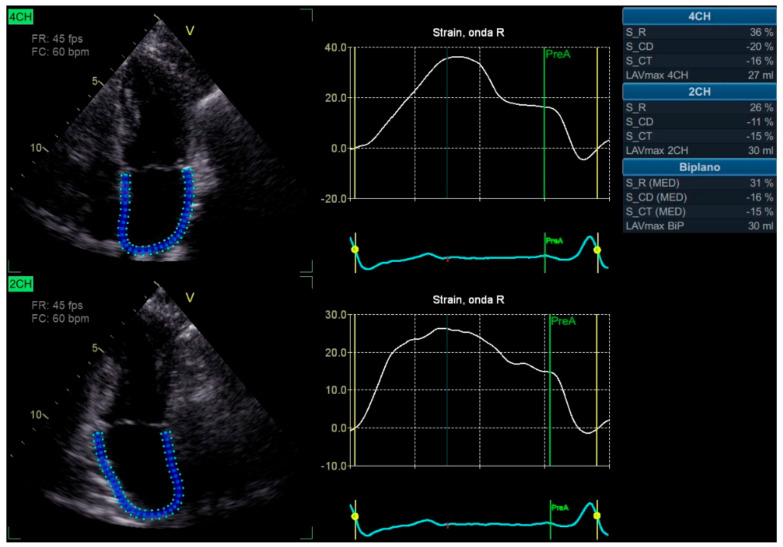
Speckle tracking of left atrial strain. 2CH—apical two chambers view; 4CH—apical four chambers view; LAVmax—left atrial maximum volume; S_CD—left atrial strain in conduct phase; S_CT—left atrial strain in contraction phase; S_R—left atrial strain in reservoir phase.

STE allows for assessing these strain parameters with remarkable accuracy, providing a detailed picture of the LA’s functional status. However, due to the technical challenges inherent in measuring LAS, such as the difficulty delineating the atrial walls in the far field of transthoracic imaging, these parameters have not yet become routine in clinical practice. Additionally, variability between different ultrasound machines further complicates the standardization of LAS measurements [20]. Figure 3 shows an example of LAS.

While there is considerable variation in the measurements of LAS due to differences in study design, patient populations, and imaging technologies, general reference values for LA function have been reported in the literature. For example, the reservoir function of LA has been observed to average approximately 39.4% [95%, confident interval (CI) 38.0–40.8%] across 40 studies [21]. The conduit function has an average value of 23.0% (95%, CI 20.7–25.2%), as reported in 14 studies [21]. Finally, the booster function has an average value of 17.4% (95%, CI 16.0–19.0%), as reported in 18 studies [21].

Despite the technical challenges, LAS has attracted considerable interest in clinical research due to its potential to provide valuable prognostic and diagnostic information, particularly concerning AF, a prevalent arrhythmia associated with significant morbidity and mortality [22]. A reduction in LAS is a common finding in patients with AF and is associated with atrial structural remodeling, including fibrosis, loss of compliance, and reduced contractile function [23,24,25]. However, it is necessary to consider that LASr also reflects the contractile function of the left ventricle [19]. These structural alterations impair the LA’s capacity to effectively regulate pressure and volume changes, thereby contributing to the pathogenesis of AF [24,25].

The available evidence indicates that reduced LAS may serve as an early indicator of AF onset, recurrence, and progression. Specifically, lower baseline LAS in patients with paroxysmal AF is associated with an increased likelihood of developing persistent AF, reflecting progressive atrial fibrosis and dysfunction [26,27]. This indicates that LAS may serve as a valuable predictor of AF and may assist in identifying patients at elevated risk of disease progression [28].

In the context of AF management, LAS has been demonstrated to predict outcomes following therapeutic interventions, including catheter ablation and antiarrhythmic drug therapy [22]. The results of several studies have demonstrated that patients with higher pre-ablation LAS tend to have better procedural outcomes and lower recurrence rates [22]. This suggests that LAS could serve as a useful biomarker for guiding treatment decisions [22,29]. This makes LA strain a promising tool for predicting AF recurrence and evaluating the effectiveness of interventions to restore sinus rhythm.

In addition to its role in AF, LAS has been demonstrated to be a valuable tool for evaluating other cardiac pathologies, particularly those that involve structural heart disease. For example, patients with severe organic mitral regurgitation exhibit abnormalities in LA reservoir function, with impaired peak longitudinal strain correlating with worse surgical outcomes [30]. Similarly, patients with mitral stenosis demonstrate impaired conduit function, which reflects the reduced capacity of the LA to fill the left ventricle [31].

As the technology and techniques for measuring LAS improve, STE will likely become a more widely adopted tool in clinical cardiology. The potential of LAS as a biomarker for identifying patients at high risk of AF and other arrhythmias and monitoring the progression of atrial remodeling over time is a topic of ongoing research. Incorporating LAS assessment into routine clinical practice may ultimately facilitate the personalization of treatment strategies for patients with AF and other atrial disorders. This could inform decisions regarding rhythm control, anticoagulation therapy and interventional procedures.

Given its predictive value and ability to provide a detailed assessment of LA function, LAS holds promise as an essential component of future cardiovascular care. It offers predictive insights and monitoring capabilities for patients with atrial arrhythmias and other structural heart diseases.

## 3. Atrial Fibrillation: Not Just a Question of Enlargement

**Figure 4:**
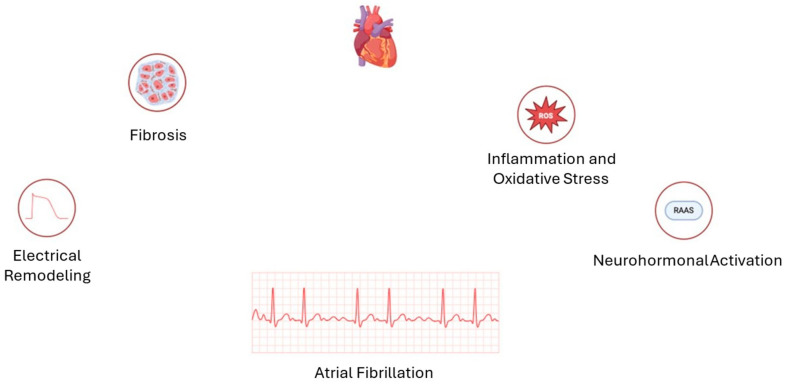
The most important mechanism of pathogenesis of atrial fibrillation.

AF is not merely a consequence of atrial enlargement; it is a complex condition encompassing structural, electrical, and biochemical remodeling within the atrial myocardium. Although atrial dilation is frequently observed in chronic AF, this enlargement is more a consequence than a cause. It arises from underlying pathological changes that progressively compromise atrial function and contribute to the arrhythmia’s persistence. A central element of the pathophysiology of AF is atrial fibrosis, a structural remodeling process characterized by excessive deposition of extracellular matrix proteins within the atrial walls [32]. Fibrotic tissue reduces the compliance and elasticity of the atria, impairing their reservoirs and contractile functions. Furthermore, it disrupts electrical conduction pathways, creating conduction delays and re-entry circuits that sustain the arrhythmia [33,34]. The fibrotic areas act as non-conductive regions, promoting disorganized electrical activity and thus perpetuating AF by generating chaotic and irregular impulses. In addition to structural remodeling, AF is driven by electrical remodeling, whereby ion channel function and calcium handling contribute to the atrial myocardium’s propensity for rapid and irregular firing. Alterations in calcium dynamics promote triggered activity through spontaneous sarcoplasmic reticulum calcium release, leading to ectopic beats and further arrhythmic activity [35]. Ion channel remodeling, including the downregulation of specific potassium and sodium channels, reduces the atrial refractory period, thereby facilitating the formation of re-entrant circuits and increasing the susceptibility of the atrium to the development of AF episodes [36]. Over time, these electrical changes become self-reinforcing, a phenomenon that is often summarized as “AF begets AF”. With each episode of AF, the atrial myocardium undergoes further structural and electrical changes, increasing the likelihood of future episodes and promoting the transition from paroxysmal (intermittent) to persistent and permanent forms of AF [37]. Figure 4 summarizes the most important mechanism of the pathogenesis of AF.

It is becoming increasingly clear that AF is a systemic disease that is influenced by several factors, including inflammation, oxidative stress, and neurohormonal activation. The elevation of inflammatory markers, such as C-reactive protein and interleukin-6, in patients with AF, suggests that inflammation plays a role in both the initiation and progression of AF. This is thought to occur by promoting atrial fibrosis and oxidative damage to cardiac cells [38,39]. Oxidative stress, an imbalance between reactive oxygen species (ROS) and antioxidants, further damages atrial tissue and contributes to fibrosis and ion channel dysfunction, thereby exacerbating AF. Furthermore, neurohormonal factors, including the activation of the renin–angiotensin–aldosterone system (RAAS), have been identified as contributing factors in the progression of AF. RAAS activation promotes fibrosis and increases atrial pressure and volume overload, thereby contributing to further structural remodeling [40].

This complex interplay between structural, electrical, and biochemical factors has led to a shift in perspective regarding AF, moving away from the traditional view that the condition is primarily caused by atrial enlargement. Instead, there is a greater recognition of the pivotal roles played by metabolic, autonomic, and systemic influences. Given these mechanisms underlying atrial dysfunction, diagnostic approaches, such as LAS, that can detect early alteration of atrial function play an important role.

## 4. Role of Physical Exercise in Atrial Dysfunction

Physical exercise causes morphological and functional changes to the heart, so much so that there is a phenomenon called ‘athlete’s heart’ [41]. The LA is also affected; physical effort causes an enlargement of the LA due to increased pressure in the left ventricle. Some observational studies have shown that in athletes, regardless of the type of physical exercise, there is an enlargement in the dimensions of the LA (2-dimensional and volume) [42,43]. However, atrial volumes normalized for total heart volume do not differ between athletes and controls, indicating that LA enlargement is proportional to total heart volume enlargement. A retrospective study by D’Andrea et al. demonstrated that power exercise was associated, albeit with an enlargement of the LA, with an improvement in atrial function expressed as LAS [44]. LA enlargement has raised concerns about the risk of AF. Sport-related AF may occur in a middle-aged male athlete with a history of long-term regular endurance sport practice, especially one involved in high-endurance training [45]. Given these premises, there is a relationship between LA function and increased left ventricular filling pressures, with different results if the pressure increase is acute or chronic.

## 5. Role of Pharmacological and Exercise Stress Echocardiography in Left Atrial Strain

SE has traditionally been used to evaluate known or suspected chronic coronary syndrome (CCS), where stress-induced ischemia leads to new or worsening regional wall motion abnormalities (RWMAs), with excellent accuracy [46,47,48]. However, SE is now recognized as a valuable tool for assessing a broader range of conditions beyond ischemic heart disease, including systolic and diastolic HF, non-ischemic cardiomyopathy, valvular heart disease, pulmonary hypertension, athletes’ hearts, congenital heart disease, and in patients post-heart transplantation [13]. Generally, SE aims to create hemodynamic conditions that reveal structural or functional cardiac abnormalities that remain occult at rest and, at the same time, match patients’ symptoms with a particular cardiac condition. This could be done both with physical exercise and with pharmacological stressor administration. Exercise is preferred for most SE applications because it preserves the body’s natural and complex electromechanical response and provides crucial information about the patient’s functional capacity. In contrast, pharmacological stress testing does not fully mimic the complex hemodynamic and neurohormonal effects of exercise, including psychological motivation and responses from the nervous, pulmonary, and circulatory systems, as well as skeletal muscle. Thus, for the scope of this review, we will refer only to exercise and dobutamine SE (ESE and DSE, respectively).

**Figure 5:**
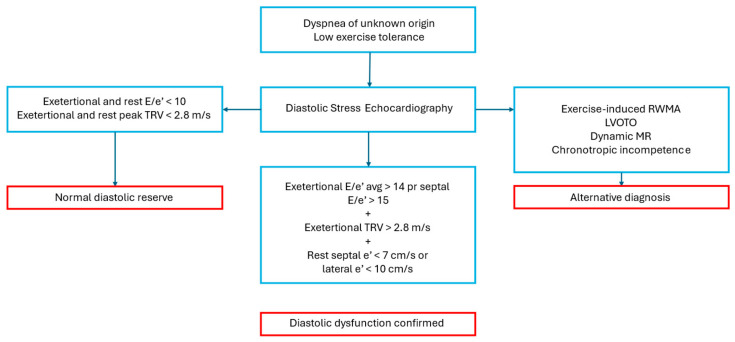
Indication and interpretation of diastolic stress echocardiography. LVOTO—left ventricular outflow tract obstruction; MR—mitral regurgitation; RWMA—regional wall motion abnormalities; TRV—tricuspid regurgitation velocity. Blue: diagnostic pathway; Red: diagnosis.

Diastolic SE is primarily used to evaluate left ventricular (LV) diastolic function reserve and detect elevated LV filling pressures. This is one of the most relevant non-ischemic indications for SE, as it is crucial for the evaluation of patients with unexplained dyspnea or subclinical diastolic dysfunction, such as those with diabetic cardiomyopathy or arterial hypertension [49]. It is important in patients with suspected HFpEF and borderline diastolic abnormalities at rest, as a non-invasive alternative to cardiac catheterization [13,15]. The presence of diastolic dysfunction in all these conditions is strictly linked to atrial structural and functional abnormalities and AF. Therefore, the timely detection of latent diastolic dysfunction is pivotal because it could lead to the initiation of therapies that might potentially reduce the burden of atrial arrhythmias [50]. The preferred method for diastolic SE is exercise on a supine bicycle, allowing continuous Doppler recording during the test to assess exercise-induced diastolic function reserve. Of note, in diastolic SE, low-workload exercise and low doses of dobutamine are usually sufficient to derive enough information for clinical management and, thus, are often suitable for patients with limited exercise capacity. Traditionally, Doppler parameters such as mitral E and A velocity (the latter is detectable only during sinus rhythm), E/A ratio, e’ velocity, E/e’ ratio, and systolic pulmonary artery pressure (SPAP) constituted the most powerful indicators for diastolic function analysis. Those parameters are recorded at baseline, during low-level and peak exercise, and in recovery. Recordings are taken from an apical four-chamber view over 5–10 cardiac cycles [13]. In healthy middle-aged individuals, the E/e’ ratio typically remains stable during exercise, as mitral inflow and annular velocities increase proportionally. This reflects a normal diastolic response. Conversely, if latent diastolic dysfunction exists, there is an exaggerated increase in mitral E velocity due to the rapid rise in LA pressure, but minimal change in e’ velocity during exercise, reflecting LV stiffness. The increase in E/e’ ratio and/or SPAP during exercise correlates with elevated LV end-diastolic pressure, as confirmed by invasive methods [13]. Figure 5 shows how to interpret a diastolic SE, according to expert consensus [13,51]. In the Heart Failure Association Pretest Assessment, Echocardiographic and Natriuretic Peptide Score, Functional Testing in Case of Uncertainty, and Final Aetiology (HFA-PEFF) diagnostic score for HFpEF, suggested by the European Society of Cardiology (ESC) expert consensus, a stress-induced average E/e’ > 15 counts as 2 points if considered alone and 3 points when associated to exercise-induced tricuspid regurgitation (TR) velocity > 3.4 m/s [15]. Markers of poor outcomes include an exercise E/septal e’ ratio over 13, limited changes in diastolic velocities, and exercise-induced pulmonary hypertension with SPAP ≥ 50 mmHg [13].

This approach, although valid, has some limitations. First, similar to what occurs in the study of diastolic function at rest, in diastolic SE, there is a “grey zone” between physiological values and what is considered definitively pathological. Moreover, the evaluation of diastolic parameters is even more complicated in patients with AF during the SE, due to the variability of the cardiac cycle and the usually elevated heart rate during ESE and DSE [52]. These limitations may at least in part be overcome using advanced echocardiographic techniques, such as atrial strain by STE. Indeed, as already mentioned, LAS is a relatively simple technique that detects atrial and diastolic dysfunction with excellent sensitivity and accuracy. Several studies have demonstrated that performing LAS is feasible during ESE and DSE and could provide clinicians with relevant diagnostic and prognostic information, particularly in the context of AF and HFpEF (Table 1). The basic principle for the utility of LAS during stress tests is that in initial phases, subtle forms of LA dysfunction, LA volume, and functional abnormalities could be absent at rest but are potentially detectable during stress as a reduced functional LA reserve [18]. In contrast, in some patients, abnormalities present at rest can be normalized by stress administration, eliciting an atrial functional reserve not utilized at rest and better outcomes [11].

Zagatina et al. conducted a multicenter observational study involving 3042 patients who had undergone SE for known or suspected CCS, divided into three groups based on the history of AF: none (group 1), paroxysmal (group 2), and permanent (group 3). The analysis showed that LAS in the reservoir phase (LASr) progressively decreased from groups 1 to 3, both at rest (group 1 = 26.0 ± 8.5%, group 2 = 23.2 ± 11.2%, and group 3 = 8.5 ± 6.5%, *p* < 0.001) and at peak stress (group 1 = 26.9 ± 10.1, group 2 = 23.8 ± 11.0, and group 3 = 10.7 ± 8.1%, *p* < 0.001). There was a linear inverse relationship between left atrial volume index (LAVi) and LASr, both at rest and during stress, both in ESE (n = 252, at rest, r = −0.387, *p* < 0.001, at peak stress: r = −412, *p* < 0.001) and pharmacological stress (n = 234, at rest: r = −0.409, *p* < 0.001, at peak stress: r = −0.269, *p* < 0.001). Therefore, this study demonstrates that atrial dysfunction at rest and during stress, defined as LASr < 24%, is more frequent and severe in patients with AF than in patients with sinus rhythm, even when the arrhythmia is absent during the test, with worse values in patients with permanent AF compared to those with paroxysmal AF. Moreover, the impairment of LASr during stress was related to more severe signs of pulmonary congestion quantified using lung ultrasound (LUS) B-lines. Lastly, this work highlighted the importance of performing LAS testing during SE, since it led to a reclassification of LA function relative to the rest evaluation (from normal to abnormal, or vice-versa) in 25.9% of cases, although performed only in 16% of the study population [11].

More recently, Prota et al. demonstrated a high feasibility and success rate for LAS during pharmacological SE in 252 patients with CCS. Moreover, although most patients showed a “normal” atrial response during SE with LASr enhancement at stress peak, with values ≥ 24%, some patients developed an abnormal response to stressors with LASr impairment coupled to LAVi and LUS B-line increase. Moreover, the study showed an inverse linear correlation between LASr and LAVi (r = −0.304, *p* < 0.001 at rest; r = −0.289, *p* < 0.001 at peak stress) and between LUS B-lines and LASr at peak stress (r = −0.234, *p* < 0.001). Most importantly, the results revealed an inverse relationship between the New York Heart Association (NYHA) functional class and LASr, both at rest and during SE (r = −0.159, *p* < 0.01 and r = −0.263, *p* < 0.001, respectively). Taken together, these findings highlight the importance of atrial dysfunction, particularly during physical activity, in the pathophysiology of pulmonary congestion. This and exercise limitation are key clinical elements in patients with AF and/or HFpEF [12].

Yoshii et al. retrospectively analyzed 74 consecutive hypertrophic cardiomyopathy (HCM) patients with EF > 50% who had undergone ESE. They found that LASr at peak workload was significantly associated with new-onset AF during follow-up in AF-naïve patients (HR 1.08, 95% CI 1.01–1.18, *p* = 0.027). Indeed, peak LASr ≤ 15.5% was predicted with a sensitivity of 55.6% and specificity of 91.8% [area under the curve (AUC) 0.71] new-onset AF. The event-free survival rate for the occurrence of new-onset AF was lower in patients with a peak LASr ≤ 15.5% than in those with a peak LASr > 15.5% (44.4% vs. 91.8% at 2 years, log-rank *p* < 0.001). Moreover, lower peak LASr was found in the subset of patients with low exercise tolerance (<75 W) (31.2 ± 15.3 vs. 24.7 ± 13.6%, *p* = 0.033) [53].

Cheng et al. prospectively performed LAS in 100 subjects with dyspnea (74 had HFpEF and 26 had non-cardiac dyspnea). They found an interesting, statistically significant, inverse relationship between stress peak LAS in the conduit phase (LAScd) and both exercise post-capillary wedge pressure (PCWP) (r = −0.659; *p* < 0.001) and ΔPCWP (difference between rest and exercise PCWP) (r = −0.707, *p* < 0.001), invasively measured during right heart catheterization (RHC). Several echocardiographic parameters were associated with ΔPCWP, but exercise LAScd was the only one with a correlation coefficient > 0.7. Moreover, they also examined the ability of exercise LAScd and other traditional echocardiographic criteria (LAVi > 34 mL/m^2^, E/e’ > 15, septal e’ < 7 cm/s) to distinguish between HFpEF and non-cardiac dyspnea and LAScd had the largest area-under-the-curve (AUC 0.69, 95% confidence interval [CI], 0.548–0.831), with a cut-off value of 14.25%, providing a sensitivity of 0.64 and a specificity of 0.68. Therefore, adding exercise LAScd to conventional echocardiographic criteria seems to slightly improve the diagnostic accuracy for HFpEF, a condition strongly related to AF [54].

The HFpEF Stress Trial prospectively enrolled 75 patients with exertional dyspnea and echocardiographic signs of diastolic dysfunction (E/e’ > 8), who underwent simultaneous rest and ESE and right heart catheterization (RHC). They found that LA compliance, defined as LASr/E/e’ ratio, was decreased in HFpEF patients diagnosed invasively during RHC, both at rest (2.0 vs. 3.2, *p* < 0.001) and during exercise-induced stress (1.4 vs. 2.6, *p* = 0.004), compared to individuals with non-cardiac dyspnea. Similarly, HFpEF patients diagnosed non-invasively according to their HFA-PEFF scores (≥5 points) showed impaired LA compliance at rest (1.7 vs. 2.8, *p* < 0.001) and during exercise stress (1.3 vs. 2.2, *p* = 0.022). Interestingly, both LA compliance at rest (HR 1.86 95% CI 1.11–3.13, *p* = 0.019) and LASr at rest (HR 1.09, 95% CI 1.02–1.16, *p* = 0.008) were predictors of cardiovascular hospitalizations independently of a history of atrial fibrillation. However, this study does not support the additional value of exercise stress for the assessment of LA compliance, probably due to technical difficulties caused by the deterioration in image quality during SE [55].

In a similar study, Harada et al. enrolled 225 HFpEF patients and 262 controls with non-cardiac dyspnea who had undergone ergometry ESE to assess LA function. HFpEF diagnosis was based on the HFA-PEFF algorithm or exercise right heart catheterization. STE evaluation was feasible both at rest and during stress testing (performed in 95% and 89% of patients, respectively), showing that HFpEF patients had significantly lower LAS values and LA compliance at rest (LASr 31.8 ± 13.2 vs. 19.7 ± 11.3, *p* < 0.0001; LAScd 16.1 ± 8.9 vs. 10.6 ± 6.3, *p* < 0.0001; LASct 16.7 ± 7.8 vs. 12.2 ± 6.9, *p* < 0.0001; LA compliance 3.7 ± 2.1 vs. 1.7 ± 1.3, *p* < 0.0001) and during exercise (LASr 38.9 ± 15.5 vs. 22.7 ± 13.1, *p* < 0.0001; LAScd 15.4 ± 9.9 vs. 11.1 ± 7.1, *p* < 0.0001; LASct 26.4 ± 12.8 vs. 15.9 ± 10.2, *p* < 0.0001; LA compliance 3.8 ± 1.81. vs.  ± 1.2, *p* < 0.0001) compared to non-cardiac dyspnea. Both LA compliance and peak LASr had the strongest diagnostic ability to differentiate HFpEF from non-cardiac dyspnea (AUC 0.87, 0.83–0.90, *p* < 0.0001 and AUC 0.82, 0.67–0.91, *p* < 0.0001, respectively), outperforming the exercise E/e’ ratio (DeLong *p* = 0.005). The optimal LA compliance cut-off value was 2.2%, providing 81% sensitivity and 85% specificity for HFpEF diagnosis, while the optimal peak LASr cut-off value was 31% with 78% sensitivity and 70% specificity [56].

**Figure 6:**
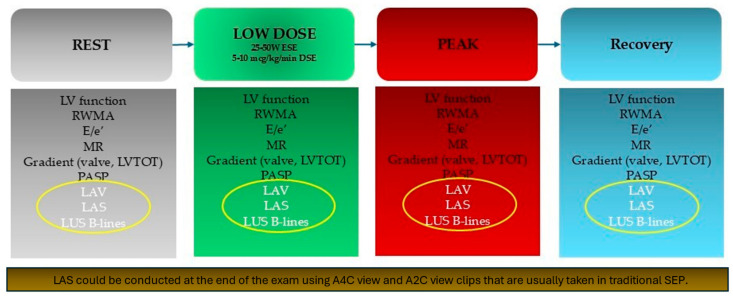
Stress echocardiography protocol includes evaluation of left atrial function and pulmonary congestion. A2C—apical 2-chamber; A4C—apical 4-chamber; DSE—dobutamine stress echocardiography; ESE—exercise stress echocardiography; LAS—left atrial strain; LAV—left atrial volume; LV—left ventricle; LVOT—left ventricle outflow tract; LUS—lung ultrasounds; MR—mitral regurgitation; PASP—pulmonary artery systolic pressure; RWMA—regional wall motion abnormalities; SEP—stress echocardiography protocols.

In conclusion, these data suggest that traditional SE protocols could easily be enriched by the inclusion of LA morphological and functional evaluation, as well as the assessment of LUS B-lines in an ABCDE + SE protocol (Figure 6) [57]. This is particularly indicated in patients undergoing SE due to dyspnea of unknown origin, since atrial dysfunction is a key pathophysiological element in HFpEF and LA evaluation during stress may allow the detection of an incipient LA myopathy that predisposes the development of AF and pulmonary congestion. Among all LAS reserve parameters during SE, peak-LASr is probably the most simple and useful in predicting AF onset and recurrence. Indeed, LASr is the most extensively studied among LAS measurements in the context of rest and stress echocardiography. We believe a peak LASr cut-off value of 24% should be used, while a more stringent cutoff of <15.5% has greater specificity, but at the cost of lower sensitivity. The 24% threshold aligns well also with the findings of most studies focused on LAS in rest echocardiography (Table 2) [7].

## 6. What Do Guidelines on AF Say? Actual Evidence and Future Perspectives

ESC guidelines for the management of AF have recently been published. Although numerous studies have been published on the predictive role of decrease in atrial function detected as a modification of atrial strain value, the guidelines do not include this echocardiographic parameter [1]. However, the guidelines may come very close to considering diastolic function as a determinant to be evaluated in patients with FA. The guidelines introduce an approach based on the AF-CARE algorithm [57,58]. Comorbidities are associated with the recurrence and progression of AF. Managing comorbidities is also central to the success of other aspects of care for patients with AF, with evidence available for hypertension, HF, diabetes mellitus (DM), obesity, and sleep apnea, along with lifestyle changes that improve physical activity and reduce alcohol intake. A correlation between the risk factors mentioned and numerous studies has demonstrated an alteration in atrial strain.

- Arterial hypertension: Miljković et al., in a cross-sectional study that considered 180 patients with systemic arterial hypertension, showed that LAS represents a predictive factor for diastolic HF in patients with systemic arterial hypertension (*p* < 0.0001) [1].
- HF: Barki et al., in a prospective study of 85 consecutive patients with reduced, moderately reduced, and HFpEF, demonstrated that in acute HF of any LV ejection fraction, LA dynamics are highly predictive of rehospitalization compared to nt-pro-brain natriuretic peptide (*p* = 0.01) [59].
- DM: Thiele et al. in a prospective, placebo-controlled exploratory study, evaluated how the use of empagliflozin 10 mg daily associated with an improvement in glycated hemoglobin was associated with a significant improvement in LA after 3 months of treatment, as assessed by an increase in LASr and LASct values (from 26.4 ± 8.0% to 29.0 ± 7.4%; *p* = 0.011 and from 10.9 ± 5.7% to 12.5 ± 6.0%; *p* = 0.008) compared to placebo [60].
- Obesity: Aga et al. showed, in a prospective study that enrolled 77 obesity patients compared with 46 non-obese controls, there was significantly decreasing LA function compared with non-obese individuals (LASr 32.2% ± 8.8% vs. 39.6% ± 10.8%, *p* < 0.001; LAScd 20.1% ± 7.5% vs. 24.9% ± 8.3%, *p* = 0.001; LASct 12.1% ± 3.6% vs. 14.5% ± 5.5%, *p* = 0.005). One year after bariatric surgery, LASr improved (32.1% ± 8.9% vs. 34.2 ± 8.7%, *p* = 0.048). In the multivariable linear regression analysis, body mass index (BMI) was associated with LASr, LAScd, and LASct (β = −0.34, CI −0.54 to −0.13; β = −0.22, CI −0.38 to −0.06; β = −0.10, CI −0.20 to −0.004) [61].
- Sleep apnea: there are no studies into the relationships between LAS and sleep apnea
- Alcohol intake: Alam AB et al., in a randomized trial, enrolled 503 participants. They showed that higher alcohol consumption (increased by one drink per day) was associated with lower LASct (−0.44% [95% CI, −0.75 to −0.14]) [62].

However, current studies evaluate LAS in patients with comorbidities with a resting echocardiogram. By analogy, assessing changes in LAS through SE execution could represent an important prognostic factor in patients with comorbidities. Therefore, large population studies would be needed to evaluate the predictive role of SE in patients with comorbidities at risk of AF.

In addition, an important prognostic role, in the construction of risk scores, could be played by artificial intelligence (AI). Recently, Sannino and Delgado evaluated the role of AI in the study of atrial function as an LAS value [63]. Their editorial evaluates the study performed by Carluccio et al. It is the first study that includes LASr in a machine learning algorithm to define clusters of LV diastolic dysfunction and evaluate the prognostic implications [27]. They evaluated the predictive value of a novel machine learning-based algorithm that includes conventional echocardiographic variables and LASr. The machine learning-based approach was trained in 864 patients with HF and sinus rhythm and validated in 189 outpatients with HF. By using an LASr cut-off value of 19%, a total of three specific clusters of LV diastolic dysfunction were identified [27]. This study represents a milestone in the use of AI in the evaluation of LAS. In addition to being able to use AI in echocardiogram images at rest, in the future nothing precludes using these protocols for images acquired during stress echocardiography. These images could be useful for developing predictive and risk scores of AF.

## 7. Conclusions

Currently, SE represents an important diagnostic and prognostic tool in CCS. In clinical practice, SE is used to evaluate valvular defects and the evaluation of diastolic function. LAS represents one of the echocardiographic parameters of diastolic function, but it is also a parameter of atrial function. Therefore, its diagnostic and prognostic role could benefit clinical practice for diseases dependent on an LA dysfunction, such as AF. Large clinical studies are needed to demonstrate LAS dysfunction’s diagnostic and predictive role during SE, paying attention to categories of people, such as athletes, where LAS seems to improve despite an enlargement of the LA.

## Figures and Tables

**Table 1:** Most important studies into the use of left atrial strain during stress echocardiography.

Author	Design	Population	Results
Zagatina et al. [11]	Multicenter prospective observational study	3042 pts undergone SE for CCS (group 1: no AF; group 2: paroxysmal AF; group 3: permanent AF)	LAS during SE performed in 16% of cases; peak LASr group 3 < LASr group 2 < LASr group 3 (group 1 = 26.9 ± 10.1, group 2 = 23.8 ± 11.0, and group 3 = 10.7 ± 8.1%, *p* < 0.001).
Prota et al. [12]	Single-center prospective observational study	252 pts undergoing pharmacological SE for CCS	LAS during SE performed in 95.5% of cases; inverse linear correlation between peak LASr and LAVi (r = −0.289, *p* < 0.001); inverse linear correlation between LUS B-lines peak LASr (r = −0.234, *p* < 0.001); inverse correlation between peak LASr and NYHA class (r = −0.263, *p* < 0.001, respectively).
Yoshii et al. [53]	Single-center retrospective study	74 HCM pts with EF > 50% undergoing ESE	Peak LASr associated with new-onset AF (HR 1.08, 95% CI 1.01–1.18, *p* = 0.027); peak LASr ≤ 15.5% predicted with a sensitivity of 55.6% and specificity of 91.8% (AUC 0.71) new-onset AF; lower peak LASr correlated to low exercise tolerance (<75 W) (31.2 ± 15.3 vs. 24.7 ± 13.6%, *p* = 0.033).
Cheng et al. [54]	Single-center prospective observational study	100 pts with dyspnea (74 HFpEF and 26 NCD)	Inverse correlation between peak LAScd and PCWP (r = −0.659; *p* < 0.001) and ΔPCWP (r = −0.707, *p* < 0.001); peak LAScd < 14.25% detect HFpEF with 64% sensitivity and 68% specificity (AUC 0.69).
Backhaus et al. [55]	Single-center prospective observational study	75 pts with exertion dyspnea and rest E/e’ > eight undergoing ESE	LASr/E/e’ ratio during stress decreased in HFpEF patients diagnosed invasively (1.4 vs. 2.6, *p* = 0.004) compared to individuals with NCD. LASr/E/e’ ratio during stress decreased in HFpEF patients diagnosed non-invasively (1.3 vs. 2.2, *p* = 0.022).
Harada et al. [56]	Single-center retrospective study	487 pts undergone ESE (225 HFpEF pts + 262 controls with NCD)	LAS during SE performed in 89% of cases; exercise LAS and LASr/E/e’ ratio lower in HFpEF compared to NCD; exercise LASr/E/e’ ratio and peak-LASr had the strongest diagnostic ability to differentiate HFpEF from NCD (AUC 0.87, 0.83–0.90, *p* < 0.0001 and AUC 0.82, 0.67–0.91, *p* < 0.0001, respectively); exercise LASr/E/e’ ratio < 2.2%, 81% sensitivity and 85% specificity for HFpEF diagnosis.

AF—atrial fibrillation; AUC—area under the curve; CCS—chronic coronary syndrome; CI—confidence intervals; ΔLASct%—left atrial strain contraction reserve during stress; ΔPCWP—difference between rest and stress PCWP; EF—ejection fraction; ESE—exercise stress echocardiography; HCM—hypertrophic cardiomyopathy; HFpEF—heart failure with preserved ejection fraction; HR—hazard ratio; LAS—left atrial strain; LAScd—left atrial strain conduit phase; LASct—left atrial strain contraction phase; LASr—left atrial strain reservoir phase; LAVi—left atrial volume index; LUS—lung ultrasound; METS—metabolic equivalent of task; NCD—non-cardiac dyspnea; NYHA—New York Heart Association; PCWP—post-capillary wedge pressure; Pts—patients; SE—stress echocardiography.

**Table 2:** Abnormal values of LAS parameters during SE.

Author	Sample Size	LAS Parameters During SE and Abnormal Values
Zagatina et al. [11]	3042 pts	Peak LASr < 24%
Prota et al. [12]	252 pts	Peak LASr ≤ 24%
Yoshii et al. [53]	74 pts	Peak LASr ≤ 15.5%
Cheng et al. [54]	100 pts	Peak LAScd < 14.25%
Harada et al. [56]	487 pts	Peak LASr < 31% (protocol 1) or <33.4% (protocol 2)Exercise LASr/E/e’ ratio < 2.2% (protocol 1) or <2% (protocol 2)

LAS—left atrial strain; LAScd—left atrial strain conduit phase; LASr—left atrial strain reservoir phase; pts—patients; SE—stress echocardiography.
